# Baseline CD3+CD56+ (NKT-like) Cells and the Outcome of Influenza Vaccination in Children Undergoing Chemotherapy

**DOI:** 10.3389/fimmu.2021.690940

**Published:** 2021-06-29

**Authors:** Evelin A. Leibinger, Gábor Pauler, Noémi Benedek, Tímea Berki, István Jankovics, Richard McNally, Gábor Ottóffy

**Affiliations:** ^1^ Division of Pediatric Hematology and Oncology, Department of Pediatrics, University of Pécs Medical School, Pécs, Hungary; ^2^ Institute of Mathematics and Informatics, University of Pécs, Pécs, Hungary; ^3^ Department of Immunology and Biotechnology, University of Pécs Medical School, Pécs, Hungary; ^4^ Department of Virology, National Center for Epidemiology, Budapest, Hungary; ^5^ Population Health Sciences Institute, Newcastle University, Newcastle, United Kingdom

**Keywords:** influenza vaccines, pediatrics, immunosuppression, natural killer T-cells, lymphocyte subsets, cellular immunity, CD4+ T-cells

## Abstract

**Background:**

In children undergoing chemotherapy yearly influenza vaccination is recommended by treatment protocols. We investigated the relationship between cellular immunity and the antibody response to inactivated influenza vaccines.

**Methods:**

25 patients (age: 2-18 years) undergoing chemotherapy for different malignancies participated in our study. Flow cytometric detection of peripheral blood lymphocyte subpopulations together with hemagglutination inhibition antibody titers were measured before and 21-28 days after vaccination. We examined the ratio and total numbers of CD3+, CD4+, CD8+ T cells, activated helper (CD3+CD4+CD25^low^), regulatory (CD3+CD4+CD25^high^), naive (CD3+CD45RA+) and memory (CD3+CD45RO+) T cells, CD56+NK, and CD3+CD56+ (NKT-like) cells. Relationships between specific antibody responses (seroprotection, seroconversion, geometric mean titer (GMT), geometric mean fold increase (GMFI)) and the ratios and counts of lymphocyte subpopulations were evaluated using one-way ANOVA and the paired sample t test after dichotomization according to age-related reference values.

**Results:**

Patients with CD4+ lymphocyte levels in the normal age-specific range showed significantly better response regarding postvaccination GMT elevation for H1N1 and H3N2 strains (97.52 *vs*. 19.2, p=0.019, 80 *vs*. 14.43, p=0.021, respectively). GMFI results were significant only against B strain (2.69-fold *vs*. 1.23-fold, p=0.046). Prevaccination CD3+CD56+ (NKT-like) cells above predicted values according to age showed significant associations both in postvaccination GMT elevation (H1N1: 75.11 *vs*. 14.14, p=0.010; H3N2: 62.18 *vs*. 11.22, p=0.012; B: 22.69 *vs*. 6.67, p=0.043) and GMFI against all three strains (H1N1: 3.76-fold *vs*. 1.06-fold, p=0.015; H3N2: 2.74-fold *vs*. 1, p=0.013; B: 2.57-fold *vs*. 1, p=0.008). By one-way ANOVA, we found a positive relation between absolute lymphocyte cell count above 1000/µl and the postvaccination GMT elevation against H3N2 (12.81 vs. 56.56, p=0.032), and GMFI regarding H1N1 (1.22-fold *vs*. 3.48-fold, p=0.044).

**Conclusions:**

In addition to verifying the predictive value of absolute lymphocyte count above 1000/µl, our results suggest an association between NKT-like cell counts and the specific antibody response against all three investigated influenza strains in highly immunosuppressed patients. Furthermore, prevaccination CD4+ lymphocyte levels in the normal age-specific range may influence seroresponse.

## Introduction

Chemotherapy induced cytopenias cause prolonged immunosuppression in patients with malignancies resulting in increased susceptibility for pathogens. An annually recurring potential risk is infection with influenza. It can cause severe illness and complications as well as delays in treatment regimens thus threatening the outcome of cancer treatment (1, 2). The recommended vaccination offers the possibility to prevent infection and its complications (3).

Furthermore, investigating the immune response and factors that might be associated with successful immunization of such an immunocompromised population gives us a unique opportunity to evaluate the underlying immunological mechanisms and find targets for vaccine improvement.

In a previous study we evaluated the immune response and safety of concomitant trivalent-inactivated vaccine for seasonal influenza viruses (H1N1, H3N2 and B), and monovalent-inactivated vaccine for the 2009 pandemic influenza A virus in children undergoing chemotherapy (4). In agreement with several other studies (5–9), we verified that a higher baseline absolute lymphocyte count may enhance the immune response to influenza vaccination. A remaining question is which specific lymphocyte subpopulation could be responsible for this enhancement.

In recent years cellular, especially T cell responses gained increasing importance in influenza vaccine research and development (10–12). One such area of ongoing research are natural killer (NK) T cells, also called as NKT-like cells, a heterogeneous group of T lymphocytes that confer natural immunity. These cells have the phenotypic characteristics of both T cells and NK cells and function as a link between innate and adaptive immunity. NKT-like cells include a wide group of innate T lymphocyte populations, such as γδT cells, and CD1- and MR-1-restricted T cells which have an important immunoregulatory function. Unlike the major histocompatibility complex (MHC)-restricted T cells, these cells possess a restricted repertoire of T cell receptors, perform rapid effector responses and recognize a limited selection of non-peptide molecules, presented by the MHC class I-like molecule CD1d. Upon activation, NKT cells rapidly release large quantities of multiple cytokines and chemokines capable of boosting adaptive immune responses. They are capable of stimulating a wide array of immune cells that enhance vaccine-mediated immune responses (13).

Induction of NKT cells with a special adjuvant has resulted in improved immune responses in several animal models (14) of influenza vaccination. NKT cells show similarity in mammals so induction of these cells during vaccination might be promising in humans as well. Furthermore, based on animal investigations, some authors suggest that NKT cell induction might have a role also in the treatment of influenza infection (15).

Every finding in humans that correlates with results of animal investigations supports a similar role of NKT cells in humans and leads us closer to improved prevention or treatment of influenza virus infection.

In our study we investigated the relationship between cellular immunity and the response to influenza vaccines in children receiving chemotherapy with different types of malignancies.

## Materials and Methods

### Patient Characteristics

Twenty-five consecutively treated patients (11 males and 14 females) were enrolled in our study during the influenza season periods of 2016/2017 and 2017/2018. Median age was 6 years (range 2 to 18 years). The underlying diseases were hematologic malignancies in the majority of participants (19 patients), while the remaining 6 cases had various solid tumors. All patients had been receiving chemotherapy at the Division of Pediatric Hematology and Oncology of the University of Pécs within 1 month before vaccination, and all continued to receive scheduled chemotherapy afterwards. Nine patients were undergoing maintenance therapy and sixteen children were receiving intensive cytostatic treatment (**Table 1**).

**Table 1:** Patient characteristics.

N°	Age	Sex	Malignancy	Treatment	IgG level (g/l)	Day 0 antibody titers		
						H1N1	H3N2	B
1	5	F	ALL (precursor B-cell)	maintenance	9.70	5	80	5
2	16	M	ALL (precursor B-cell)	maintenance	10.70	40	160	20
3	4	F	ALL (precursor B-cell)	intensive	7.10	5	5	5
4	5	F	ALL (precursor B-cell)	maintenance	6.32	5	80	5
5	12	F	ALL (precursor B-cell)	maintenance	5.11	80	20	5
6	3	M	ALL (precursor T-cell)	intensive	5.12	160	5	5
7	4	M	ALL (precursor T-cell)	maintenance	7.18	160	5	5
8	3	M	ALL (precursor B-cell)	intensive	5.66	5	40	5
9	4	M	ALL (precursor B-cell)	maintenance	10.30	5	40	5
10	11	M	neuroblastoma	intensive	5.10	5	5	5
11	7	M	ALL (precursor B-cell)	intensive	5.85	5	5	5
12	7	F	PNET (grade IV)	intensive	4.27	40	5	5
13	3	F	ALL (precursor B-cell)	intensive	5.08	5	5	5
14	14	F	AML	maintenance	10.00	40	5	160
15	8	F	gliosarcoma	intensive	9.09	160	320	80
16	9	M	Langerhans cell histiocytosis	intensive	8.35	80	40	40
17	12	M	osteosarcoma	intensive	4.60	40	40	5
18	2	F	myelomonocytic leukemia	intensive	4.52	5	5	5
19	17	F	mixed germ cell tumor	intensive	14.00	40	40	20
20	4	F	ALL (precursor B-cell)	intensive	6.53	5	5	5
21	17	F	ALL (precursor B-cell)	intensive	3.19	20	5	5
22	18	F	ALL (precursor B-cell)	maintenance	4.12	80	20	5
23	5	F	ALL (precursor B-cell)	intensive	5.91	5	40	5
24	16	M	ALL (precursor T-cell)	maintenance	6.85	5	5	5
25	2	M	embryonal rhabdomyosarcoma	intensive	3.05	5	5	5

ALL, acute lymphoblastic leukemia; AML, acute myeloid leukemia; PNET, primitive neuroectodermal tumor.

Exclusion criteria were the following: recent history of influenza vaccination, confirmed influenza infection prior to vaccination, history of egg allergy, administration of other vaccines during the study period. One patient was eventually excluded from the study due to lack of adherence to follow-up visits.

### Study Design, Vaccine and Schedule

The protocol for vaccination was designed in accordance with the recommendations of the Infectology Department of the Hungarian National Healthcare Advisory Board, based on data received from the Hungarian National Center of Epidemiology (16, 17).

The Institutional Review Board of the University of Pécs authorized the protocol for this study (3672.316-2954/2010). Sample collections were performed concurrently with the routine blood sampling involved in cancer treatment. Written consent was obtained from the parents of each child.

In both seasons, a whole-virion, trivalent, inactivated, adjuvanted influenza vaccine (3Fluart - Fluart Innovative Vaccines Ltd., Budapest, Hungary) was in use for vaccination. Each preparation contained aluminium phosphate gel adjuvant and 6 µg haemagglutinin of each strain per 0.5 ml. Strains were selected according to the recommendations of the World Health Organization for the northern hemisphere for influenza vaccine composition for respective seasons: A/Hong Kong/4801/2014 (H3N2)-like, B/Brisbane/60/2008-like, A/California/7/2009 (H1N1)pdm09-like (A/California/7/2009, NYMC X-181) in 2016/2017 and A/Michigan/45/2015 (H1N1)pdm09-like in 2017/2018.

Fifteen patients were younger than 10 years old, and therefore received 0.25 ml of the vaccine, while ten participants received 0.5 ml of the vaccine.

In patients receiving maintenance treatment, vaccine was given without discontinuation of therapy. During intensive treatment, vaccination was scheduled in all patients 2-3 days before the next cytostatic treatment according to the recommendations of the Hungarian National Healthcare Advisory Board.

### Sample Collection and Serological Analysis

Blood samples were taken from each participant before vaccination and 28 days afterwards, mostly *via* central venous catheter for measurements of hemagglutination inhibition (HI) titer, immunoglobulin level, white blood cell count and flow cytometric analysis.

Three millilitres of serum were separated by centrifugation, then immediately frozen and stored at -80°C until the laboratory measurements on HI antibody titers were performed. Titers against the virus strains were measured by hemagglutination inhibition with chicken red blood cells following recommended procedures (18). All serologic tests were done at a single central laboratory (Department of Virology, National Center of Epidemiology, Budapest, Hungary). Each pair of sera were tested in complete duplicate series on the same day with the use of identical reagents.

The geometric mean HI antibody titer (GMT), seroprotection (defined as a titer level of ≥1:40) rate, seroconversion (defined a ≥4-fold increase in titer from baseline or a postvaccination titer level of ≥1:40 if the baseline titer was undetectable) rate, and GMT fold increase (GMFI: GMT ratio of postvaccination titer to prevaccination titer) were calculated.

### Multiparametric Flow Cytometry Analysis of Phenotypes

**Figure 1:**
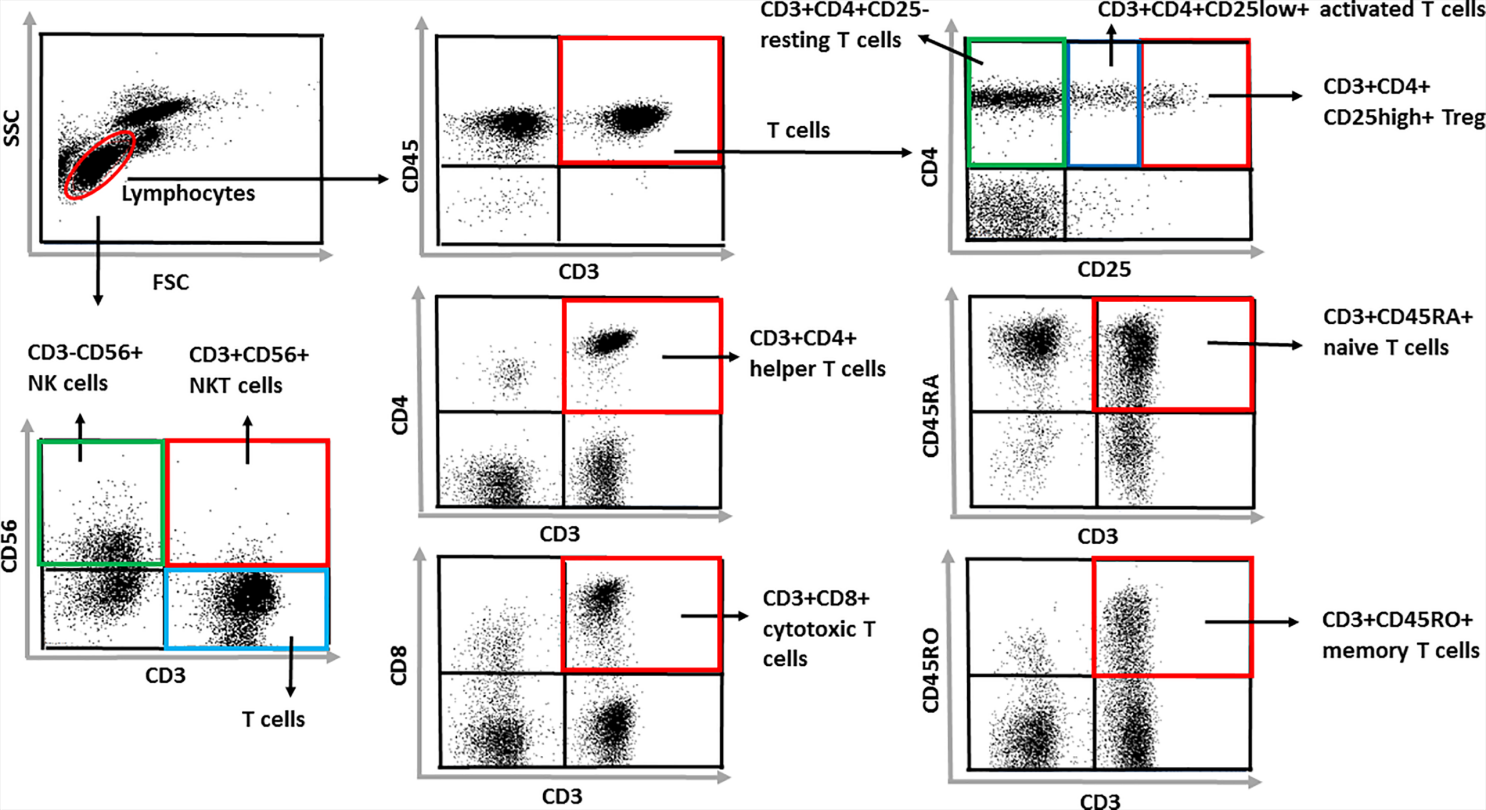
Gating strategies. At least 10,000 cells were collected in the lymphocyte gate and analyzed. CD3+, CD4+, CD5+, CD8+ T cells, CD3+CD4+CD25- “resting T_helper_ cells”, CD3+CD4+CD25^low^ “activated” and, CD3+CD4+CD25^high^ “regulatory” T cells were detected and their absolute cell numbers were calculated. We followed naive CD3+CD45RA+ and CD3+CD45RO+ memory T cell percentages and absolute cell counts, CD3+CD56+ NKT-like and CD56+ NK cell ratio and absolute cell counts. CD3/CD45 positive cells were further analyzed for their CD4, CD8 and CD25 expression. Naive and memory T cells were detected with simultaneous CD3/CD45RA/CD45RO staining, NK cells and NKT cells were analyzed from the lymphocyte gate based on their CD3/CD56 positivity. For each of the gated cell populations the percentages of cells were analyzed.

The following anti-human monoclonal antibodies were used: anti-CD3-FITC, anti-CD8-PE, anti-CD4-PerCP, anti-CD56-PECy5, anti-CD25-PE, anti-CD45RA-PE and anti-CD45RO-PerCP (all from BD Biosciences). The 50 µl blood sample (EDTA) was incubated with the appropriate antibody cocktails for 30 minutes on ice, washed twice in phosphate buffered saline and fixed with 1% paraformaldehyde prior flow cytometric analysis using a FACSCalibur cytometer with CellQuestPro software. At least 10,000 cells were collected in the lymphocyte gate and analyzed. CD3+, CD4+, CD5+, CD8+ T cells, CD3+CD4+CD25- “resting T_helper_ cells”, CD3+CD4+CD25^low^ “activated” and, CD3+CD4+CD25^high^ “regulatory” T cells were detected and their absolute cell numbers were calculated. We followed naive CD3+CD45RA+ and CD3+CD45RO+ memory T cell percentages and absolute cell counts, CD3+CD56+ NKT-like and CD56+ NK cell ratio and absolute cell counts. CD3/CD45 positive cells were further analyzed for their CD4, CD8 and CD25 expression. Naive and memory T cells were detected with simultaneous CD3/CD45RA/CD45RO staining, NK cells and NKT cells were analyzed from the lymphocyte gate based on their CD3/CD56 positivity. For each of the gated cell populations the percentages of cells were analyzed. Gating strategies are presented in **Figure 1**.

### Adverse Reactions and Medical Conditions

Baseline assessments on day 0 consisted of gathering demographic data, medical history and physical examination. We examined each patient on a weekly basis by physical and laboratory (complete blood count) examination during the follow-up period, until the final blood sampling was obtained for serologic study. Possible vaccine-related adverse events were monitored closely. On follow-up visits, standard medical history and medications administered since the last visit were summarized, and physical examination was performed prior to blood sample collection. Safety variables were recorded during follow-up visits for patient history and physical examination.

### Statistical Analysis

Data were analyzed using one-way ANOVA and paired sample t test using the package SPSS 20.0. F-tests for main effects were obtained using one-way ANOVA (weighted method). For one-way ANOVA analyses we divided our sample of patients into two groups based on age-related reference values and where these were not available, statistically determined cut-off values for each cellular parameter to investigate whether a certain cell count or ratio might be related to better serologic response.

We handled pre- and postvaccination HI titers in two ways.

We analyzed GMT levels at pre- and postvaccination: due to skewed distributions, analyses were performed on the natural log of HI titer and arithmetic means were converted to GMT with exponential transformation.

We calculated values of GMFI between pre- and postvaccination: as it was discussed earlier (19) that in a population which is not entirely seronegative before immunization, prevaccination HI titer levels have significant influence on postvaccination titers, we examined the ratio of post-/prevaccination GMTs.

Due to the methodology of serologic analyses, negative specimens (undetectable titer levels) were assigned a nominal value of 5 for the purposes of statistical analysis. For each test, a p-value < 0.05 was considered significant.

We analyzed day 0 results for the following parameters: absolute lymphocyte count, total CD3+ T, CD4+ T_h_ and CD8+ T_c_ cell count, CD4/CD8 ratio, CD3+CD45RA+ naive and CD3+CD45RO+ memory T cell count and naive/memory T cell ratio. Furthermore, we followed T_reg_ cell (CD3+CD4+CD25^High^) and activated T_helper_ (CD3+CD4+CD25^Low^) cell counts, CD3+CD4+CD25^High/Low^ ratio, CD3+CD56+ NKT-like and CD56+ NK cell counts; and compared them with postvaccination HI titers (postvaccination GMT) or with the GMFI between day 0 and day 28. We conducted these studies (postvaccination GMT and GMFI) separately for each virus strain: H1N1pdm09-like, H3N2-like and B/Brisbane strain.

## Results

### Antibody Responses

GMTs, seroprotection and seroconversion rates were calculated on day 0 and on day 28. GMTs of antibodies to different influenza virus strains are listed in **Table 2**. All postvaccination GMTs were higher than prevaccination GMTs, regarding H1N1, H3N2 and B/Brisbane strains, and examining GMFI, increases between them were significant. Seroprotection and seroconversion rates according to virus strains are shown in **Table 3**.

**Table 2:** GMT and GMFI values on day 0 (prevaccination) and day 21-28 (postvaccination) according to virus strains.

	H1N1	H3N2	B
**Prevaccination GMT [95% CIs]**	16.93 [9.59, 29.88]	15.58 [9.01, 26.93]	7.79 [5.20, 11.66]
**Postvaccination GMT [95% CIs]**	30.84 [15.73, 60.45]	23.78 [11.59, 48.77]	11.55 [6.27, 21.26]
**GMFI [95% CIs]**	1.88 [1.10, 3.23]	1.58 [1.04, 2.41]	1.54 [1.06, 2.24]
**p values of GMFI**	0.023	0.032	0.025

GMT, Geometric Mean Titer; GMFI, Geometric Mean Fold Increase. 95% CIs are expressed as lower/upper bounds of GMT or GMFI values.

**Table 3:** Pre- and postvaccination rates for seroprotection^†^ and seroconversion^‡^ by virus strains.

	H1N1 n/N (%)	H3N2 n/N (%)	B n/N (%)
**Prevaccination seroprotection**	11/24 (46)	9/24 (38)	3/24 (13)
**Postvaccination seroprotection**	12/24 (50)	10/24 (42)	5/24 (21)
**Seroconversion**	6/24 (25)	4/24 (17)	3/24 (13)

^†^Seroprotection defined as a HI titer ≥40.

^‡^Seroconversion defined as at least four-fold rise in titer postvaccination.

### Flow Cytometry Results According to Age-Related Reference Values

According to the age-matched reference intervals provided by Huenecke et al. (20) we observed that in most cases, total lymphocyte count, CD3+, CD4+, CD8+ and CD56+ counts were under the lower limit of age-related intervals. CD4/CD8 ratio was within the normal range in 52% of our patients. Interestingly, percentages of CD3CD25+ activated T cells and CD4CD25+ activated T_h_ cells were in normal ranges in all of our patients. For certain subpopulations however, no age-dependent reference ranges were available. Since intervals were not available for CD3+CD56+ NKT cells, we used the predicted values and found that 50% of our patients had results under this cut-off value. Descriptive results are summarized in **Table 4**.

**Table 4:** Flow cytometry results describing the baseline cellular parameters (absolute counts and percentages) compared to age-related normal values.

Baseline cellular parameters	Number and percentage of patients with results within age-related normal values	
Total lymphocyte count	3/25	12%
CD3 (n/µl)	4/25	16%
CD4/CD8 ratio	13/25	52%
CD4 (n/µl)	8/25	32%
CD8 (n/µl)	15/25	60%
CD3CD25 activated T cells (%)	25/25	100%
CD4CD25 activated T_h_ cells (%)	25/25	100%
CD4CD25^High^ T_reg_ cells (%)	*no reference available*	
CD3CD45RA naive T cells (%)	14/25	56%
CD3CD45RO memory T cells (%)	23/25	92%
CD3CD25 activated T cells (n/µl)	*no reference available*	
CD4CD25 activated T_h_ cells (n/µl)	*no reference available*	
CD4CD25^High^ T_reg_ cells (n/µl)	*no reference available*	
CD3CD45RA naive T cells (n/µl)	*no reference available*	
CD3CD45RO memory T cells (n/µl)	*no reference available*	
CD56 NK (%)	12/25	48%
CD56 NK (n/µl)	2/25	8%
CD3CD56 NKT (%)	*no reference available*	
CD3CD56 NKT (n/µl) ^†^	12/24	50%

^†^As reference ranges were not available for each child, respective age-dependent predicted values were used for CD3+CD56+ NKT-like cells.

### Relationship of Lymphocyte Subpopulations and the Immune Response

**Supplementary Table 1**. shows the absolute cell numbers and percentages analyzed using flow cytometry and the ratios of certain cells. By assessing specific cell population counts, we examined the relations between lymphocyte subpopulations and their ratios and the outcome of vaccination. The associations of prevaccination T cells and GMT and GMFI by different strains is shown in **Tables 5** and **6**. As mentioned before, a p-value < 0.05 was considered significant for each test.

**Table 5:** Relations between different prevaccination cellular parameters and postvaccination GMT by virus strains. Significant results are written in bold.

			H1N1		H3N2		B	
	Group^†^	n	GMT [95% CIs]	p	GMT [95% CIs]	p	GMT [95% CIs]	p
Lymphocyte count, n/μl	< 1000	14	21.01 [9.98, 44.22]	0.168	12.81 [5.45, 30.09]	**0.032**	8.62 [4.41, 16.83]	0.249
	≥ 1000	10	52.77 [13.72, 202.90]		56.56 [17.08, 187.33]		17.41 [4.96, 61.02]	
Lymphocyte count, n/μl	< Norm	21	32.81 [15.54, 69.3]	0.626	23.59 [10.56, 52.7]	0.951	13.02 [6.55, 25.9]	0.293
	≥ Norm	3	20 [0.64, 626.08]		25.2 [0.7, 907.91]		5 [5, 5]	
CD3+ absolute count, n/μl	< Norm	20	29.28 [13.86, 61.85]	0.729	20.7 [9.32, 45.98]	0.384	11.49 [5.87, 22.46]	0.966
	≥ Norm	4	40 [2.32, 690]		47.57 [3.02, 749.6]		11.89 [0.75, 187.4]	
CD4+ absolute count, n/μl	< Norm	17	19.2 [9.8, 37.61]	**0.019**	14.43 [6.84, 30.45]	**0.021**	8.49 [4.87, 14.82]	0.105
	≥ Norm	7	97.52 [9.8, 483.56]		80 [15.94, 401.54]		24.38 [3.87, 153.51]	
CD4/CD8 ratio	< Norm	12	18.88 [7.91, 45.05]	0.134	16.82 [6.69, 42.3]	0.329	10 [4.23, 23.64]	0.635
	≥ Norm	12	50.4 [17.04, 149.03]		33.64 [9.94, 113.8]		13.35 [4.9, 36.35]	
CD3+CD56+ (NKT-like) absolute count, n/μl	< Pred	12	14.14 [6.75, 29.62]	**0.010**	11.22 [5.19, 24.25]	**0.012**	6.67 [3.53, 12.60]	**0.043**
	≥ Pred	11	75.11 [24.73, 228.12]		62.18 [19.05, 202.96]		22.69 [7.42, 69.39]	

^†^Grouping strategies: ‘Norm’ refers to the lower limit of age-related reference intervals, whereas ‘Pred’ refers to the age-specific predicted values of CD3+CD56+ cells. GMT, Geometric Mean Titer; GMFI; Geometric Mean Fold Increase, n, number of patients.

**Table 6:** Relations between different prevaccination cellular parameters and GMFI by virus strains. Significant results are written in bold.

			H1N1		H3N2			B		
	Group^†^	n	GMFI [95% CIs]	p		GMFI [95% CIs]	p		GMFI [95% CIs]	p
Lymphocyte count, n/μl	< 1000	14	1.22 [0.69, 2.17]	**0.044**		1.56 [0.78, 3.13]	0.926		1.16 [0.92, 1.46]	0.061
	≥ 1000	10	3.48 [1.26, 9.59]			1.62 [1.01, 2.60]			2.30 [0.96, 5.47]	
Lymphocyte count, n/μl	< Norm	21	1.87 [1.01, 3.46]	0.935		1.64 [1.02, 2.65]	0.677		1.64 [1.07, 2.51]	0.377
	≥ Norm	3	2 [0.36, 11.19]			1.26 [0.47, 3.4]			1 [1, 1]	
CD3+ absolute count, n/μl	< Norm	20	1.57 [0.93, 2.64]	0.114		1.57 [0.95, 2.58]	0.902		1.41 [1.04, 1.92]		0.294
	≥ Norm	4	4.76 [0.26, 86.51]			1.68 [0.59, 4.83]			2.38 [0.15, 37.48]	
CD4+ absolute count, n/μl	< Norm	17	1.39 [0.79, 2.44]	0.062		1.44 [0.81, 2.58]	0.477		1.23 [0.96, 1.57]		**0.046**
	≥ Norm	7	4 [1.05, 15.19]			2 [1.18, 3.38]			2.69 [0.75, 9.63]	
CD4/CD8 ratio	< Norm	12	2.38 [1.02, 5.52]	0.386		1.78 [0.96, 3.31]	0.581		1.5 [0.77, 2.91]		0.887
	≥ Norm	12	1.5 [0.68, 3.28]			1.41 [0.73, 2.75]			1.59 [0.99, 2.55]	
CD3+CD56+ (NKT-like) absolute count, n/μl	<Pred	12	1.06 [0.59, 1.89]	**0.015**		1 [0.51, 1.97]	**0.013**		1 [1, 1]		**0.008**
	≥ Pred	11	3.76 [1.5, 9.41]			2.74 [1.77, 4.23]			2.57 [1.21, 5.5]	

^†^Grouping strategies: ‘Norm’ refers to the lower limit of age-related reference intervals, whereas ‘Pred’ refers to the age-specific predicted values of CD3+CD56+ cells. GMT, Geometric Mean Titer; GMFI; Geometric Mean Fold Increase, n, number of patients.

By one-way ANOVA, we did not find a relation between age-related baseline absolute lymphocyte counts and postvaccination GMT and GMFI against the three influenza strains. However, after comparing the immune response of patients with absolute lymphocyte cell counts below or above 1000/µl, these results suggested a positive association between higher cell counts and vaccine response. We found a positive relation with the postvaccination GMT elevation against H3N2 (12.81 *vs.* 56.56), and GMFI regarding H1N1 (1.22-fold *vs*. 3.48-fold).

Regarding CD4+ lymphocytes, we expected better immune responses in patients with CD4+ cell counts in the normal age-specific range and they showed significantly better results in postvaccination GMT elevation for H1N1 and H3N2 strains (97.52 *vs*. 19.2 and 80 *vs*. 14.43, respectively). However, GMFI results were significant only against B strain.

Analyses of age-related predicted values of CD3+CD56+ cells showed significant relations both with postvaccination GMT elevation and GMFI against all three strains. Patients with CD3+CD56+ counts above the predicted threshold had better responses in GMT after vaccination (H1N1: 75.11 *vs*. 14.14; H3N2: 62.18 *vs*. 11.22; B: 22.69 *vs*. 6.67). GMFI results were significant regarding all strains as well (H1N1: 3.76-fold *vs*. 1.06-fold, H3N2: 2.74-fold *vs*. 1, B: 2.57-fold *vs*. 1).

Considering that age-specific references were not available for all measured cellular parameters, we tried to establish cut-off values based on statistical methods.

We compared the role of T_reg_ cells and found that the ratio of regulatory/activated T cells (CD3+CD4+CD25^High^ *vs*. CD3+CD4+CD25^Low^) above 10% suggested a negative association with postvaccination GMT results in H3N2 strain (42.02 *vs*. 10.72, respectively). Patients with an absolute T_reg_ (CD3+CD4+CD25^High^) cell count <10/µl showed a tendency to have higher GMFI (2.46-fold *vs*. 1.16-fold) against H3N2 strain. The activated T cell (CD3+CD4+CD25^Low^) count did not show relations with postvaccination GMT or GMFI with any of the strains.

Analyses of CD4/CD8 ratio, CD3+CD45RA+ naive and CD3+CD45RO+ memory T cell counts, naive/memory T cell ratio, CD3+, CD8+, and CD56+ NK cell counts did not show significant associations with immune response against any of the strains.

### Adverse Reactions

We recorded no local adverse reactions (injection site induration, erythema, swelling or warmth). No medical intervention was necessary. No vaccine related serious adverse event was observed. There were no breakthrough influenza infections.

## Discussion

Myelosuppressive effects of therapeutic protocols are considerably diverse depending on the underlying malignancy and the cytotoxic agents administered. In hematological malignancies immunosuppressive effects persist during treatment and recovery can take more than 12 months after cessation of therapy. Acute lymphocytic leukemia patients have decreased lymphocyte counts in all major groups compromising both humoral and cellular immunity possibly affecting preexisting humoral protection resulting from childhood vaccinations or previous infections. Total leukocyte and lymphocyte counts were found to be comparable to healthy children after 1 year (21). However, flow cytometry investigations demonstrated that recovery of specific subsets might take much longer (22), CD3+, CD4+, CD8+ cells were affected most (23–25). Recovery of naive T cells to age-related normal levels was found to take 1-6 months (22, 26), which can be explained by the ability of the thymus to produce new naive cells in childhood.

CD4+ cell counts were found to be persistently low in a long term investigation of pediatric ALL patients, with decreases both in CD3+CD45RO and CD3+CD45RA subsets (21, 26). Significant decrease in the ratio of CD4+ and CD8+ cells was reported in multiple studies during and after chemotherapy treatment (27). NK cells did not show significant differences after different time points of treatment completion (21).

Solid tumor patients show much faster reconstitution of immune status. Lower immunoglobulin levels mostly recovered within the first six months. Severely decreased CD4+ T cell count resulting in lower CD4+/CD8+ ratio, persisted briefly after therapy compared to leukemia patients. Total T lymphocytes recovered within six to twelve months (28, 29). NK cells were found to be increased immediately after treatment and normalized within 6 months (28).

As we expected, our patients had markedly lower cell counts in most measured subpopulations so we anticipated a modest immune response against influenza vaccination. A recently published multicenter immunogenicity trial with a trivalent influenza vaccine of the same manufacturer and composition as the one used in our study gave us the opportunity to compare our results to healthy children. In two age groups (3-11 years and 12-18 years) postvaccination seroprotection rates varied between 80-96.67% and seroconversion rates were between 58.33-70% (30). Although in our group of patients, postvaccination GMTs were significantly higher than prevaccination values against all three strains, considerably worse seroconversion and seroprotection results were consistent with reports in immunocompromised populations.

Based on our previous results (4) and other reports on the predictive value of baseline lymphocyte counts (5, 8), we expected a positive association between the absolute lymphocyte count above 1000/µl on day 0 and HI titer changes independent of virus strains. We could reconfirm these results against H3N2 in GMT and H1N1 and B in GMFI. Analyses based on age-related normal values did not show significant associations with vaccine response.

We expected to find a positive relation between higher CD4+ (T_helper_) cell counts and the serosresponse. In healthy adults, the predictive role of absolute CD4+ T cell count has already been reported. Jürchott et al. (31) found that CD4+ T cell count along with other factors could predict seroprotection against pandemic influenza strain (A(H1N1)pdm09) with 89% accuracy. We found that baseline absolute CD4+ T cell count within the age-related interval showed a positive relation with both postvaccination GMT (H1N1, H3N2) and GMFI (B/Brisbane). Furthermore, this result seems to be consistent with findings in vaccine research regarding other immunocompromised populations such as HIV infected individuals, where the ability to mount a strong response is largely dependent on CD4+ cell count.

The most noteworthy result we recognised was the positive association of day 0 CD3+CD56+ (NKT-like) cell count above the age-specific predicted values and both postvaccination GMT and GMFI. We observed the most predictive role of these cells for the seroresponse against all strains of influenza virus.

According to Romero-Olmedo et al. (32), human peripheral blood CD3+CD56+ cells constitute a phenotypical continuum of different natural T cell subsets such as CD8+ or CD4-CD8- (double negative), γδ+ and MAIT cells. They demonstrated that both in healthy and allergic adults, CD3+CD56+ cells were actually part of all major T cell groups rather than a separate group.

Expression of CD56 has been associated with cytotoxic and immunostimulatory properties (33) and has been found on several activated cell types that also show cytotoxic properties. This finding suggests that CD56 is more of a sign of activation than a characteristic phenotypical marker.

However, we think that these results might have more importance in influenza vaccine research. CD3 and CD56 markers have been associated with NKT cells as well. Although the terminology of these cells is quite discordant (34), they are often referred to as NKT-like cells, a heterogeneous group of T cells that have been the target of extensive research regarding influenza vaccines. CD3+CD56+ (NKT-like) cells can carry out MHC-unrestricted cytotoxicity and secrete many kinds of cytokines. They confer natural immunity and play a part in helping the differentiation of helper T cells.

Several research groups have demonstrated that activation of NKT cells contribute to the humoral immune response to different microbes (35), however, most of these studies were conducted in animals. In connection with influenza vaccination, these studies demonstrated that use of glycolipid adjuvants (e.g. α-galactosylceramide) as NKT cell activators lead to better immunological response (36–38). Regarding recent investigations into the immunology of SARS-CoV-2 infection, lower percentages of CD3+CD56+ (NKT-like) cells have been observed in COVID-19 subjects when compared to healthy controls. Further comparison of patients with severe and non-severe pneumonia showed significantly lower percentages in the former group of subjects (39).

A possible theory is that the relationship between the immune response and the number of NKT-like cells in this immunosuppressed population might originate from their homing characteristics. Most of these cells migrate and reside to different tissues (40), therefore they might form a pool of mobile cells during chemotherapy. However there are only a few reports about findings that support this phenomenon (41, 42).

We would like to further investigate and confirm our results by an extended, nationwide study of the connection between prevaccination lymphocyte subpopulations, especially NKT-like cells (with more detailed identification) and the outcome of influenza vaccination in children undergoing chemotherapy.

As we expected, a modest immunogenicity was observed in our patients. These findings seemed to be in agreement with published reports.

Our study has several limitations. The patient sample was considerably heterogeneous in respect of specific hemato-oncologic diseases and types of cytostatic treatment. Statistically the sample size was quite small and no control group was established. Consequently, our results should be treated with caution. Nevertheless, we tried to compensate it with sampling mostly those patients, who received parenteral cytostatic treatment. Additionally, we used a conventional p value of 0.05.

## Conclusion

To our knowledge, we revealed for the first time some possible connections between T-lymphocyte subpopulation counts at the time of vaccination and seroresponse to influenza vaccination in immunosuppressed patients. From a practical point of view, although in a small, but highly immunosuppressed study population our investigation suggests a possible positive association between NKT-like cells and postvaccination GMT and GMFI in all three strains following influenza vaccination.

After verifying our results on a larger sample with a control group, it can outline a promising direction for research to further specify the associations of the previously mentioned cell populations and their ratios and serologic immunity. Our investigation highlights the need for age-specific reference ranges for T cell subpopulations. Moreover, should our results be verified, they could support the possible advantages of incorporating NKT cell-activating adjuvants into influenza vaccines.

## Data Availability Statement

The raw data supporting the conclusions of this article will be made available by the authors, without undue reservation.

## Ethics Statement

The studies involving human participants were reviewed and approved by Institutional Review Board of the University of Pécs, Pécs, Hungary (3672.316-2954/2010). Written informed consent to participate in this study was provided by the participants’ legal guardian/next of kin.

## Author Contributions

EL: Data curation (supporting); Writing – Original Draft Preparation (equal); Writing – Review and Editing (equal). GP: Data curation (lead); Formal analysis (lead); Investigation (equal); Visualization; Writing – Review and Editing (equal). NB: Conceptualization (equal); Data curation (equal); Investigation (equal). TB: Conceptualization (equal); Data curation (equal); Investigation (equal); Writing – Review and Editing (equal); IJ: Data curation (equal); Investigation (equal). RM: Supervision; Writing – Review and Editing (equal). GO: Conceptualization (lead); Data curation (equal); Funding acquisition; Investigation (equal); Project administration (lead); Writing – Original Draft Preparation (equal); Writing – Review and Editing (equal). All authors contributed to the article and approved the submitted version.

## Funding

Statistical work was partially financed by 2020-4.1.1-TKP2020, the Subprogram 3: “Innovation for sustainable life and environment” of “Scientific Field Excellence Program 2020” of the Hungarian Government.

## Conflict of Interest

The authors declare that the research was conducted in the absence of any commercial or financial relationships that could be construed as a potential conflict of interest.

## Abbreviations

ALL, acute lymphoblastic leukemia; AML, acute myeloid leukemia; HI, hemagglutination inhibition; GMT, geometric mean titer; GMFI, geometric mean fold increase; MHC, Major Histocompatibility Complex; MAIT, mucosa associated invariant T cells; MR-1, MHC-related molecule-1; NKT cells, natural killer T cells; PNET, primitive neuroectodermal tumor; SPSS, Statistical Package for Social Sciences; T_h_, helper T cells; T_c_, cytotoxic T cells; T_reg_, regulatory T cells.
