# Supplementary material for: Baseline CD3+CD56+ (NKT-like) Cells and the Outcome of Influenza Vaccination in Children Undergoing Chemotherapy
Source: Front Immunol. 2021 Jun 29;12:690940. doi: 10.3389/fimmu.2021.690940 (PMC8276261; doi:10.3389/fimmu.2021.690940)
Supplement: Supplementary file 1 [file Table_1.docx]

**Supplementary Table 1.** Flow cytometry results of lymphocyte subpopulation counts and ratios before (Day 0) and after vaccination (Day 28).

|  |  | **Patients** | | | | | | | | | | | | | | | | | | | | | | | | |
| --- | --- | --- | --- | --- | --- | --- | --- | --- | --- | --- | --- | --- | --- | --- | --- | --- | --- | --- | --- | --- | --- | --- | --- | --- | --- | --- |
|  | **Day** | **1** | **2** | **3** | **4** | **5** | **6** | **7** | **8** | **9** | **10** | **11** | **12** | **13** | **14** | **15** | **16** | **17** | **18** | **19** | **20** | **21** | **22** | **23** | **24** | **25** |
| **Total lymphocyte count (n/µl)** | *0* | 1300 | 1130 | 980 | 2140 | 950 | 790 | 1140 | 990 | 6810 | 430 | 1090 | 740 | 4700 | 490 | 1230 | 640 | 1400 | 620 | 880 | 860 | 320 | 90 | 1110 | 800 | 580 |
|  | *28* | 470 | 900 | 1470 | 1370 | 1020 | 730 | 950 | 850 | 2070 | 760 | 1120 | 640 | 1260 | 440 | 270 | 640 | 980 | 850 | - | 900 | 960 | 410 | 1090 | 1220 | 490 |
| **CD3 (n/µl)** | *0* | 1131 | 989 | 785 | 1834 | 700 | 750 | 1081 | 915 | 6224 | 302 | 1038 | 596 | 3624 | 368 | 1085 | 541 | 1326 | 546 | 713 | 809 | 246 | 73 | 975 | 761 | 546 |
|  | *28* | 392 | 769 | 1216 | 1241 | 766 | 699 | 902 | 756 | 1573 | 601 | 1053 | 490 | 1252 | 388 | 227 | 513 | 866 | 702 | - | 883 | 909 | 301 | 1000 | 1135 | 356 |
| **CD4/CD8 ratio** | *0* | 0.65 | 1.72 | 0.72 | 0.7 | 1.7 | 1.26 | 1.88 | 0.93 | 0.33 | 0.91 | 1.19 | 0.48 | 3.79 | 0.77 | 2.94 | 2.27 | 1.96 | 0.6 | 1.95 | 0.5 | 0.89 | 1.04 | 1.55 | 0.47 | 0.7 |
|  | *28* | 0.97 | 1.78 | 0.52 | 0.96 | 1.85 | 1.37 | 1.65 | 1.15 | 0.78 | 1.56 | 1.32 | 0.47 | 4.13 | 0.93 | 2.04 | 1.65 | 1.68 | 0.39 | - | 0.45 | 0.33 | 0.26 | 1.73 | 0.33 | 0.99 |
| **CD4 (n/µl)** | *0* | 436 | 563 | 301 | 706 | 436 | 392 | 691 | 420 | 1423 | 138 | 513 | 189 | 2829 | 157 | 742 | 342 | 847 | 192 | 473 | 271 | 109 | 35 | 482 | 230 | 190 |
|  | *28* | 188 | 456 | 379 | 570 | 504 | 391 | 538 | 391 | 662 | 357 | 541 | 150 | 989 | 183 | 131 | 292 | 517 | 184 | - | 271 | 214 | 61 | 520 | 264 | 161 |
| **CD8 (n/µl)** | *0* | 670 | 328 | 420 | 1010 | 256 | 312 | 368 | 450 | 4345 | 151 | 431 | 393 | 747 | 202 | 252 | 151 | 433 | 322 | 243 | 538 | 122 | 34 | 311 | 484 | 269 |
|  | *28* | 194 | 256 | 731 | 592 | 272 | 285 | 327 | 340 | 849 | 230 | 411 | 322 | 239 | 195 | 64 | 177 | 308 | 477 | - | 603 | 647 | 232 | 301 | 800 | 162 |
| **CD3CD25 activated T cells (%)** | *0* | 12.8 | 19 | 16 | 4.3 | 16.3 | 23.8 | 3 | 23.8 | 8.7 | 17.5 | 24.3 | 13 | 15.2 | 17 | 13.4 | 19 | 15.7 | 8 | 12.5 | 20.5 | 18.5 | 25.1 | 11.5 | 18 | 17.1 |
|  | *28* | 16.6 | 23.6 | 7.9 | 5.6 | 18.1 | 23.1 | 15.6 | 27 | 15.5 | 25.1 | 24.4 | 8.5 | 17.2 | 22.8 | 19.1 | 21.1 | 16.8 | 12.8 | - | 17.5 | 7.8 | 8.6 | 11.8 | 8 | 16.1 |
| **CD4CD25 activated T_h_ cells (%)** | *0* | 30.5 | 34 | 21.3 | 11.5 | 32.5 | 45 | 17.1 | 93.7 | 30.8 | 44.3 | 50 | 10.7 | 24.8 | 15 | 20.7 | 36.2 | 23 | 8 | 20.4 | 56.9 | 37.6 | 60.8 | 24.7 | 53.7 | 45 |
|  | *28* | 42 | 38 | 21.4 | 12.2 | 35 | 38.8 | 23.2 | 23 | 14.3 | 46.4 | 44.1 | 38.3 | 19.8 | 41.2 | 34.4 | 44.9 | 29 | 47.2 | - | 54.8 | 27.8 | 59.1 | 23.7 | 42.9 | 39.9 |
| **CD4CD25^High^ T_reg_ cells (%)** | *0* | 3.8 | 2.3 | 19.5 | 0.8 | 2.9 | 3.4 | 10.8 | 3.3 | 0.4 | 7.7 | 3 | 2.6 | 1.1 | 2 | 1.6 | 1.5 | 1.3 | 2.5 | 1 | 6.8 | 7.6 | 3 | 1.6 | 2.1 | 6.4 |
|  | *28* | 2.2 | 3.7 | 4 | 0.9 | 2.7 | 2.6 | 2 | 20 | 2 | 4.8 | 3.2 | 1.6 | 2.5 | 7.8 | 2.9 | 1.1 | 0.1 | 4.5 | - | 3.4 | 5.1 | 2.9 | 1.1 | 1.7 | 6.5 |
| **CD3CD45RA naive T cells (%)** | *0* | 29.9 | 47.7 | 40.5 | 55.5 | 56.7 | 63.8 | 67.4 | 60.3 | 41.4 | 32.8 | 47.4 | 39.1 | 64.2 | 29.5 | 57 | 39.4 | 69.9 | 15.5 | 44.5 | 66.7 | 478.1 | 31.7 | 49.6 | 45.6 | 41.6 |
|  | *28* | 43.7 | 39.9 | 36.2 | 61.7 | 56.9 | 62 | 63.3 | 65 | 45 | 34.3 | 45 | 38.4 | 69.3 | 37.6 | 29.7 | 26 | 53.3 | 26.9 | - | 76.4 | 32.9 | 14.6 | 58.8 | 45.2 | 26.6 |
| **CD3CD45RO memory T cells (%)** | *0* | 59.5 | 41 | 35.4 | 31.9 | 23.7 | 31.7 | 26.7 | 32 | 50.5 | 38.8 | 47.7 | 41.9 | 15.2 | 45.3 | 32.9 | 43.6 | 25.5 | 74.3 | 42.1 | 27.8 | 27.3 | 23 | 39.7 | 48.8 | 40.2 |
|  | *28* | 43.2 | 50.1 | 43.7 | 27.5 | 23.1 | 34.5 | 30.4 | 28 | 35 | 45.5 | 50.9 | 39.1 | 29.4 | 53.3 | 49.8 | 53.5 | 35.6 | 57.5 | - | 22 | 62.2 | 59.7 | 34 | 48.8 | 44.5 |
| **CD3CD25 activated T cells (n/µl)** | *0* | 144.77 | 187.91 | 125.6 | 78.86 | 114.1 | 178.5 | 32.43 | 217.77 | 541.49 | 52.85 | 252.23 | 77.48 | 550.85 | 62.56 | 145.39 | 102.79 | 208.18 | 43.68 | 89.13 | 165.85 | 45.51 | 18.32 | 112.13 | 136.98 | 93.37 |
|  | *28* | 65.07 | 181.48 | 96.06 | 69.5 | 138.65 | 161.47 | 140.71 | 204.12 | 243.82 | 150.85 | 256.93 | 41.65 | 215.34 | 88.46 | 43.36 | 108.24 | 145.49 | 89.86 | - | 154.53 | 70.9 | 25.89 | 118 | 90.8 | 57.32 |
| **CD4CD25 activated T_h_ cells (n/µl)** | *0* | 132.98 | 191.42 | 64.11 | 81.19 | 141.7 | 176.4 | 118.16 | 393.54 | 438.28 | 61.13 | 256.5 | 20.22 | 701.59 | 23.55 | 153.59 | 123.8 | 194.81 | 15.36 | 96.49 | 154.2 | 40.98 | 21.28 | 119.05 | 123.51 | 85.5 |
|  | *28* | 78.96 | 173.28 | 81.11 | 69.54 | 176.4 | 151.71 | 124.82 | 89.93 | 94.67 | 165.65 | 238.58 | 57.45 | 195.82 | 75.4 | 45.06 | 131.11 | 149.93 | 86.85 | - | 148.51 | 59.49 | 36.05 | 123.24 | 113.26 | 64.24 |
| **CD4CD25^High^ T_reg_ cells (n/µl)** | *0* | 16.57 | 12.95 | 58.7 | 5.65 | 12.64 | 13.33 | 74.63 | 13.86 | 5.69 | 10.63 | 15.39 | 4.91 | 31.12 | 3.14 | 11.87 | 5.13 | 11.01 | 4.8 | 4.73 | 18.43 | 8.28 | 1.05 | 7.71 | 4.83 | 12.16 |
|  | *28* | 4.14 | 16.87 | 15.16 | 5.13 | 13.61 | 10.17 | 10.76 | 78.2 | 13.24 | 17.14 | 17.31 | 2.4 | 24.73 | 14.27 | 3.8 | 3.21 | 0.52 | 8.28 | - | 9.21 | 10.91 | 1.77 | 5.72 | 4.49 | 10.47 |
| **CD3CD45RA naive T cells (n/µl)** | *0* | 338.17 | 471.75 | 317.93 | 1017.87 | 396.9 | 478.5 | 728.59 | 551.75 | 2576.74 | 99.06 | 492.01 | 233.04 | 2326.61 | 108.56 | 618.45 | 213.15 | 926.87 | 84.63 | 317.29 | 539.6 | 1176.13 | 23.14 | 483.6 | 347.02 | 227.14 |
|  | *28* | 171.3 | 306.83 | 440.19 | 765.7 | 435.85 | 433.38 | 570.97 | 491.4 | 707.85 | 206.14 | 473.85 | 188.16 | 867.64 | 145.89 | 67.42 | 133.38 | 461.58 | 188.84 | - | 674.61 | 299.06 | 43.95 | 588 | 513.02 | 94.7 |
| **CD3CD45RO memory T cells (n/µl)** | *0* | 672.95 | 405.49 | 277.89 | 585.05 | 165.9 | 237.75 | 288.63 | 292.8 | 3143.12 | 117.18 | 495.13 | 249.72 | 550.85 | 166.7 | 356.97 | 235.88 | 338.13 | 405.68 | 300.17 | 224.9 | 67.16 | 16.79 | 387.08 | 371.37 | 219.49 |
|  | *28* | 169.34 | 385.27 | 531.39 | 341.28 | 176.95 | 241.16 | 274.21 | 211.68 | 550.55 | 273.46 | 535.98 | 191.59 | 368.09 | 206.8 | 113.05 | 274.46 | 308.3 | 403.65 | - | 194.26 | 565.4 | 179.7 | 340 | 553.88 | 158.42 |
| **CD56 NK (%)** | *0* | 5.2 | 2.1 | 3.4 | 6.5 | 8.1 | 1.4 | 0.8 | 4.1 | 2.9 | 14.1 | 1.4 | 11.6 | 0.2 | 17.3 | 4.5 | 8.4 | 1 | 7 | 2.3 | 2 | 10.5 | 0.5 | 5.1 | 2.9 | 2.6 |
|  | *28* | 2.1 | 1.3 | 8.5 | 3.4 | 1.7 | 0.5 | 0.6 | 3 | 5 | 11.4 | 0.8 | 12.2 | 0.2 | - | 3.1 | 12.4 | 5.8 | 7.3 | - | 0.8 | - | 15.2 | 2.6 | 3.2 | 7 |
| **CD56 NK (n/µl)** | *0* | 59 | 21 | 27 | 119 | 57 | 11 | 9 | 38 | 180 | 43 | 15 | 69 | 7 | 64 | 49 | 45 | 13 | 38 | 16 | 16 | 26 | 0 | 50 | 22 | 14 |
|  | *28* | 8 | 10 | 103 | 42 | 13 | 3 | 5 | 23 | 79 | 69 | 8 | 60 | 3 | - | 7 | 64 | 50 | 51 | - | 7 | - | 46 | 26 | 36 | 25 |
| **CD3CD56 NKT (%)** | *0* | 1.2 | 8.7 | 0.6 | 1.7 | 0.9 | 7.3 | 2.3 | 13.3 | 3.5 | 1.8 | 12.8 | 0.6 | 0.3 | 5.7 | 2.8 | 6.7 | 1 | 52 | 5.5 | 1.6 | - | 7 | 1.1 | 16 | 1.3 |
|  | *28* | 0.7 | 1.4 | 1.5 | 1.4 | 0.4 | 3 | 2.8 | 3 | 1.2 | 1.3 | 0.9 | 1 | 2.2 | - | 1.4 | 1.2 | 1.4 | 5.5 | - | 1.6 | 9.1 | 1 | 1.8 | 6.1 | 0.3 |
| **CD3CD56 NKT (n/µl)** | *0* | 14 | 86 | 5 | 31 | 6 | 55 | 25 | 122 | 218 | 5 | 133 | 4 | 11 | 21 | 30 | 36 | 13 | 284 | 39 | 13 | - | 5 | 11 | 122 | 7 |
|  | *28* | 3 | 11 | 18 | 17 | 3 | 21 | 25 | 23 | 19 | 8 | 9 | 5 | 28 | - | 3 | 6 | 12 | 39 | - | 14 | 83 | 3 | 18 | 69 | 1 |
